# Improvement of antioxidant status after Brazil nut intake in hypertensive and dyslipidemic subjects

**DOI:** 10.1186/s12937-015-0043-y

**Published:** 2015-05-29

**Authors:** Grazielle V. B. Huguenin, Glaucia M. M. Oliveira, Annie S. B. Moreira, Tatiana D. Saint’Pierre, Rodrigo A. Gonçalves, Alessandra R. Pinheiro-Mulder, Anderson J. Teodoro, Ronir R. Luiz, Glorimar Rosa

**Affiliations:** 1 Institute of Heart Edson Saad, Federal Universityof Rio de Janeiro (UFRJ), Rio de Janeiro, Brazil; 2 Clinic of Atherosclerosis and Cardiovascular Disease Prevention, National Institute of Cardiology (INC), Rio de Janeiro, Brazil; 3 Chemistry Department, Pontifical Catholic University of Rio de Janeiro (PUC-Rio), Rio de Janeiro, Brazil; 4 Nutritional Biochemistry Laboratory, Federal University of Rio de Janeiro State (UNIRIO), Rio de Janeiro, Brazil; 5 Departamento de Nutrição e Dietética, Instituto de Nutrição Josué de Castro – Cidade Universitária – Ilha do Fundão, Av. Carlos Chagas Filho, 373, Prédio do Centro de Ciências da Saúde, Bloco J, 2° andar, sala 25, CEP: 21941-902 Rio de Janeiro, Brazil

**Keywords:** Brazil nut, Selenium, Serum lipids, Dyslipidemia

## Abstract

**Objectives:**

To investigate the effect of partially defatted Granulated Brazil nut (GBN) on biomarkers of oxidative stress and antioxidant status of hypertensive and dyslipidemic patients on nutrition and drug approaches.

**Methods:**

Ninety one hypertensive and dyslipidemic subjects of both genders (51.6 % men), mean age 62.1 ± 9.3 years, performed a randomized crossover trial, double-blind, placebo controlled. Subjects received a diet and partially defatted GBN 13 g per day (≈227.5 μg/day of selenium) or placebo for twelve weeks with four-week washout interval. Anthropometric, laboratory and clinic characteristics were investigated at baseline. Plasma selenium (Se), plasma glutathione peroxidase (GPx3) activity, total antioxidant capacity (TAC), 8-epi PGF_2α_ and oxidized LDL were evaluated at the beginning and in the end of each intervention.

**Results:**

GBN intake significantly increased plasma Se from 87.0 ± 16.8 to 180.6 ± 67.1 μg/L, increased GPx3 activity in 24,8 % (from 112.66 ± 40.09 to 128.32 ± 38.31 nmol/min/mL, *p* < 0,05), and reduced 3.25 % of oxidized-LDL levels (from 66.31 ± 23.59 to 60.68 ± 20.88 U/L, *p* < 0.05). An inverse association between GPx3 and oxidized LDL levels was observed after supplementation with GBN by simple model (β -0.232, *p* = 0.032) and after adjustment for gender, age, diabetes and BMI (β -0.298, *p* = 0.008). There wasn’t association between GPx3 and 8-epi PGF_2α_ (β -0.209, *p* = 0.052) by simple model.

**Conclusion:**

The partially defatted GBN intake has a potential benefit to increase plasma selenium, increase enzymatic antioxidant activity of GPx3 and to reduction oxidation in LDL in hypertensive and dyslipidemic patients.

**Trial registration:**

ClinicalTrials.gov Identifier NCT01990391; November 20, 2013.

## Introduction

Brazil nut is the food retaining the largest amount of selenium (Se) in its composition [1]. Se is a key component incorporated to selenoproteins involved in enzymatic functions of antioxidant, anti-inflammatory and thyroid hormones metabolism [2, 3]. It was demonstrated that the Brazil nut intake is able to significantly improve glutathione peroxidase (GPx) activity on individuals with low plasma Se [4]. GPx is a selenoprotein involved in the dismutation of hydroperoxides.

Reactive oxygen species (ROS) production is part of the human metabolism and is found in many physiological conditions [5]. Although, when there is an overproduction of ROS the body has an efficient antioxidant system available, which manages to control and reestablish balance. Therefore, the oxidative stress results from the imbalance of pro and antioxidants, with predominance of oxidants and consequent oxidative damage [6]. There is an association between atherosclerosis risk factors and ROS vascular production increase, since the main risk factors are genetics, age, hypertension, dyslipidemia, diabetes and tobacco use [7].

Lipoxygenases are production sources of ROS, since they catalyze the oxygen incorporation in polyunsaturated fatty acids [8]. Some lipoxygenase isoforms are related to the promotion of atherosclerosis by ROS generation and lipid oxidation, such as oxidized-LDL [9]. In healthy individuals, the amount of circulating oxidized-LDL, independently, is a predictor to atherosclerosis [10] and clinical coronary artery disease [11]; based in these situations, oxidized-LDL plasma concentration evaluation has been suggested as a risk biomarker for CVD, besides LDL-c concentrations. F2-isoprostanes are oxidized products of arachidonic acid generated from non-enzymatic reactions; they can derive from esterified fatty acids such as phospholipid membrane or circulating LDL [12]. Once formed, these compounds are structurally stable *in vivo*, circulating in its free form or remaining esterified in phospholipids; there can be found in both urine and plasma, hence considered good oxidative stress biomarkers [13].

Hypertension is one of the risk factors for acute myocardial infarction (MI), heart failure, stroke, peripheral arterial disease, aortic aneurysm and is one of the causes of chronic kidney disease [14]. Hypertension is associated to increase in oxidative stress and reduction of two antioxidant enzymes activity [15]. High levels of serum LDL-c and triglycerides, and low levels of HDL-c are also risk factors for cardiovascular disease [16] and a study presents that different types of hyperlipidemia are associated to oxidative stress [17].

Our hypothesis is based on the benefit effect related to the bioactive compounds of the Brazil nut on the management of hypertension and dyslipidemia, in order to improve antioxidant status in such individuals. The aim of this study was to investigate the effect of partially defatted Granulated Brazil nut on biomarkers of oxidative stress and antioxidant status of hypertensive and dyslipidemic patients on nutrition and drug approaches.

## Methods

### Population

One hundred and thirty-seven subjects were screened at the Clinic of Atherosclerosis and Cardiovascular Disease Prevention of the National Institute of Cardiology, Rio de Janeiro, RJ, Brazil, from September 2011 to September 2012. All the patients have been followed by a multidisciplinary team at the moment of the recruitment for the present study. Of the 137 subjects screened, 125 patients were eligible for the study. Males and females aged > 20 years with a referred diagnosis of dyslipidemia and hypertension that were taking medication for both conditions were included in the study. The exclusion criteria was food allergy to the Brazil nut; pregnancy or breastfeeding; undergoing in a low calorie diet; using dietary supplements containing antioxidant vitamins or minerals; using corticoid substances; and thyroid diseases, chronic renal failure, liver disease, cancer, rheumatic disease or systemic connective tissue disease. The Ethics Committee in Research of the National Institute of Cardiology, protocol 0316/11, approved the study, and it was registered at ClinicalTrials.gov (NCT01990391).

### Study design and dietary intervention

A randomized, placebo-controlled, double-blind crossover trial was performed. Subjects were provided either partially defatted Granulated Brazil nut or placebo along with nutritional counselling for dyslipidemia and hypertension [18, 19]. Each supplementation period lasted three months (twelve weeks) with monthly returns. After the first step of intervention, subjects underwent a washout period of four-week, based on SELGEN study [20], when did not receive any supplements. Randomization was simple, in blocks of 10 and based on a table of random numbers that were blinded from all researchers except one who encoded the bottles of supplements and had no contact with the center where the study was conducted.

Subjects were advised to use 01 standard measure containing 13 g per day (on average containing 227.5 μg of selenium) of partially defatted Granulated Brazil nut (Ouro Verde Amazônia® - Mato Grosso, Brazil). Partially defatted granulated Brazil nut was used in this study instead of Brazil nut kernel because the Granulated Brazil nut has high Se content compared to the Brazil nut (227,5 μg vs. 249,21 μg [21], respectively) and similar centesimal composition between each other, except for the total fat decrease and less calorie. In addition, Granulated Brazil nut allowed to blind the study and is already commercialized. The placebo (Mane do Brazil Indústria e Comércio Ltda, Rio de Janeiro, Brazil) was composed of flavored cassava flour (which has a nutty aroma) and was lightly stained (with natural caramel pigment) to approximate the appearance and smell of Granulated Brazil nut. Subjects were advised to consume 01 standard measure containing 10 g daily of placebo. The calculation of the diet was individualized and based on Total Energy Expenditure (TEE) formulas for men and women according to their nutritional status, age, and used the physical activity coefficient of 1.0 which is related to physical inactivity [22]. The distribution of macronutrients was balanced, and nutritional counselling followed the guidelines for hypertension and dyslipidemia [18, 19].

### Composition of the supplements

Partially defatted Granulated Brazil nut (Ouro Verde Amazônia® - Mato Grosso, Brazil) is made by the mechanical cold extraction of the extra-virgin oil of Brazil nut at atmosphere temperature. Thirteen grams of the partially defatted Granulated Brazil nut contained 64.4 kcal, 2.8 g carbohydrate, 3.4 g protein, 5.6 g total fat and 2.6 g dietary fiber. Ten grams of the placebo (flavored cassava flour) had a nutritional composition of [21]: 36.5 kcal, 8.92 g carbohydrate, 0.12 g protein, 0.03 g total fat, 0.65 g dietary fiber and 0.07 μg selenium.

### Analysis of selenium in partially defatted Granulated Brazil nut

The selenium content was analyzed according to the method described by Benicasa *et al.* [23] and adapted to use 0.3 g of sample, 6 mL of bidistilled nitric acid and 3 mL of hydrogen peroxide (both from Merck®, Germany). Samples were decomposed in a microwave oven (Provecto Analitica DGT 100 plus). The resulting solutions were transferred to polyethylene flasks and diluted to 50 mL with distilled and deionized water (MilliQ System, Millipore, USA, minimum resistivity of 18 MΩ cm). The reading of the ^77^Se isotope was performed by ICP-MS (Agilent 7500 CX series). The accuracy was evaluated by recovery tests and analysis of a dogfish liver certified reference material for trace metals (DOLT-3, National Research Council Canada standard, Canada). Recoveries of approximately 100 % were observed. The amount of Se observed in Granulated Brazil nut was 17.5 ± 0.2 μg/g, corresponding to 227.5 μg in 13 g of BNG.

### Evaluation questionnaires

A questionnaire was used to obtain information about sociodemographics characteristics, medical history, lifestyle, and the use of current medication. To assess physical activity, a previously validated questionnaire was used [24]. Patients who performed at least 150 min of moderate intensity exercise per week, according to international recommendations, were considered physically active [25].

### Anthropometric, blood pressure and laboratory measurements

Anthropometric evaluation was performed at baseline and included measurements of weight (kg), height (m), waist circumference (cm) and calculation of BMI (kg/m^2^). BMI was classified according to WHO [26].

Systolic blood pressure (SBP) and diastolic blood pressure (DBP) were evaluated using a sphygmomanometer. The SBP and DBP were measured twice by a trained professional, with a 1 min interval between the two measurements and the average value was used as the patient’s blood pressure.

Blood samples were collected after 12 h of overnight fasting, and laboratory evaluations were performed by an automated method (ARCHITECT *ci*8200, Abbott ARCHIECT®, Abbott Park, IL, USA) using commercial kits (Abbott ARCHITECT *c*8000®, Abbott Park, IL, USA). Serum triglyceride levels, total cholesterol and HDL-cholesterol (HDL-c) were evaluated. The LDL-cholesterol (LDL-c) were calculated using the Friedewald *et al.* formula [27].

Plasma Se levels were determined in plasma samples collected in NH Trace Element tubes with sodium heparin (VACUETTE®) and stored at − 20 °C until the time of analysis. The analysis were carried out in an inductively coupled plasma mass spectrometer (NexIon™ 300X, PerkinElmer, Massachusetts, USA), following the method adapted from [28]. Plasma samples (0.5 g) were added with 0.5 mL nitric acid and diluted with water to 5.0 mL final volume. In this case, most abundant ^80^Se isotope was monitored, due to the low Se concentrations found in plasma samples and the analysis was carried out in DRC mode with 0.75 mL min^−1^ of methane to circumvent the interferences. The plasma Se levels were used as a marker of adherence to the consumption of the supplement. Plasma Se was considered to be low when plasma levels were <90 μg/L [29].

The antioxidant activity of plasma glutathione peroxidase (GPx3) was determined by colorimetric assay (Cayman Inc., US) based on Paglia& Valentine method [30] with sensibility of 50 nmol/min/mL, intra-assay variation coefficient 5.7 % and inter-assay variation coefficient 7.2 %.

Plasma total antioxidant capacity (TAC) was evaluated by radical 2,2-diphenyl-1-picrylhydrazyl scan (DPPH) [31].

As oxidative stress markers 8-isoprostane (8-epi PGF2α) and oxidized LDL were assessed in plasma. Concentrations of 8-epiPGF2a in the plasma samples were determined by ELISA method [32, 33] with commercial kit (Cayman Chemicals, Ann Arbor, Michigan, USA) with sensibility of 2.7 pg/mL.

Oxidized LDL was determined by ELISA method [34, 35] with commercial kit (Mercodia, Uppsala, Sweden). Samples were diluted in 1:6400. The intra-assay variation coefficient was 6.13 % and the sensibility was 0.05 ng/mL.

### Statistical methods

The sample size calculation was performed with a pilot sample made up of the first 10 participants, considering an increase of 15 nmol/min/mL (15 %) on GPx3 activity after Brazil nut intervention. A power of 80 % and a bilateral confidence interval of 90 % were considered. The calculated sample size was 63 per group and this number was increased by 30 % (to total 81 subjects) to account for possible losses. The results were presented as the mean ± SD or median (25^th^–75^th^ percentile). The normality of the variables was investigated using the Kolmogorov-Smirnov test. To assess the intra intervention group’s (Granulated Brazil nut and placebo) effect the paired Student’s *t*-test was used, while the effect between groups was assessed by the Student’s *t*-test or Mann–Whitney test according to the distribution of variables. A significance level of p < 0.05 was considered significant. Simple and multiple linear regression models were used to analyze relationships between GPx3 activity and oxidized LDL and 8-epi PGF2α. Multiple linear regression model was adjusted for gender, age, diabetes and BMI. Statistical analyses were conducted with IBM® SPSS® Statistics software version 21 and the significance value considered was p < 0.05.

## Results

**Fig. 1:**
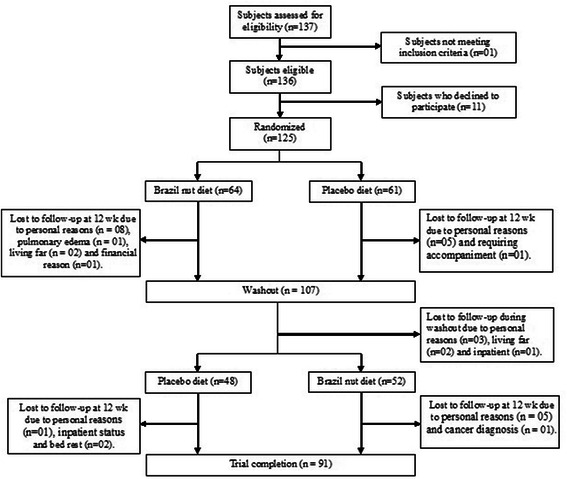
Flow chart of patients in each study phase

One hundred and twenty five patients were randomized and 91 concluded all stages of the study, resulting in a 27.2 % loss of follow up. Figure 1 shows the flow chart and losses throughout the study. The drop out in the follow-up was higher during supplementation with partially defatted Granulated Brazil nut, but there was no report of withdrawal due to the taste of the supplement. Individuals who completed the study protocol were older, with less schooling and lower serum glucose (Table 1), in relation to other parameters, there was no difference between patients who did not complete and those who completed the study.

**Table 1:** Characteristics of the group that completed the study compared to those who did not complete the study

	Completed	Did not complete	p
	(*n* = 91)	(*n* = 34)	
Age (years)	62.1 ± 9.3	56.9 ± 10.5	0.008
Male – n (%)	47 (51.6)	19 (55.9)	NS
Diabetics – n (%)	42 (46.2)	17 (50.0)	NS
Stroke – n (%)	06 (6.6)	02 (5.9)	NS
AMI – n (%)	39 (42.9)	11 (32.4)	NS
Percutaneous coronary	22 (24.2)	08 (23.5)	NS
Angioplasty – n (%)			
CABG – n (%)	29 (31.9)	08 (23.5)	NS
Angina – n (%)	36 (39.6)	16 (47.1)	NS
Time of diabetes (years)	10.6 ± 11.2	4.9 ± 7.9	NS
Time of hypertension (years)	11.8 ± 9.7	10.7 ± 8.6	NS
Time of dyslipidemia (years)	10.1 ± 9.7	9.3 ± 7.8	NS
Smokers – n (%)	02 (2.2)	02 (6.3)	NS
Alcohol use – n (%)	32 (37.2)	09 (29.0)	NS
Sedentary – n (%)	68 (74.7)	27 (79.4)	NS
Marital status (married) – n (%)	54 (60.0)	20 (62.5)	NS
Schooling – n (%)			
0 – 9 years	64 (71.1)	17 (53.1)	0.026
10 – 12 years	18 (20.0)	14 (43.8)	
≥12 years	08 (8.9)	01 (3.1)	
BMI (kg/m^2^)	28.8 ± 5.1	30.8 ± 5.5	NS
WC (cm)	100.1 ± 12.3	104.4 ± 12.5	NS
SBP (mmHg)	146.5 ± 28.7	137.9 ± 21.1	NS
DBP (mmHg)	81.8 ± 13.7	83.2 ± 13.0	NS
Glucose (mg/dL)	109.0 (90.0 - 137.0)	120.0 (102.5 - 174.0)	0.030§
Triglycerides (mg/dL)	153.0 (119.0 - 226.0)	211.0 (118.0 - 456.5)	NS §
Total cholesterol (mg/dL)	204.4 ± 61.7	217.7 ± 114.7	NS
HDL-cholesterol (mg/dL)	39.7 ± 12.3	35.8 ± 9.2	NS
LDL-cholesterol (mg/dL)	125.8 ± 54.8	116.8 ± 61.9	NS

n (%), Mean ± SD, Median (25^th^–75^th^ percentile). Chi-square test, Student’s *t*-test, § Mann–Whitney test. NS: non-significant, AMI: acute myocardial infarction, CABG: coronary artery bypass grafting, BMI: body mass index, WC: waist circumference, SBP: systolic blood pressure, DBP: diastolic blood pressure, HDL: high density lipoprotein, LDL: low density lipoprotein

A larger part of the study group was elderly (57.1 %) and there was no difference in gender distribution. Main comorbidities were diabetes and overweight and obesity, and the main events among clinical procedures were acute myocardial infarction and myocardial revascularization (Table 1). The drugs mostly used by the group were: statins (81.3 %), fibrates (33 %), oral hypoglycemic agents (48.4 %), sympatholytic agents (72.5 %), ACE inhibitors (53.8 %), diuretics (49.5 %), calcium channel blockers (40.7 %), AT1 receptor blockers (36.3 %), vasodilators (12.1 %), ASA (65.9 %).

Although all patients received treatment for dyslipidemia and hypertension, 60.4 % presented increased arterial blood pressure (systolic ≥ 140 mmHg or diastolic ≥ 80 mmHg). In relation to serum lipid concentrations 94.5 % of the patients presented some alteration on their lipid profile at the beginning of the study, being 52.7 % increased triglycerides (≥150 mg/dL), 60.4 % increased LDL-c (>100 mg/dL) and 33.3 % presented both parameters altered.

There was no change on the evolution of anthropometric and clinical parameters after twelve weeks of placebo and Brazil nut dietary interventions (data not shown), as no difference was present between diets, at the beginning or the ending periods. The same occurred for most of the biochemical parameters, however, a significant increase of HDL-c can be observed in both intervention groups (Diet + Placebo: 38.96 ± 13.30 to 41.17 ± 12.82; p < 0.05 and Diet + Granulated Brazil nut: 38.66 ± 11.91 to 40.82 ± 14.73; p < 0.05).

**Fig. 2:**
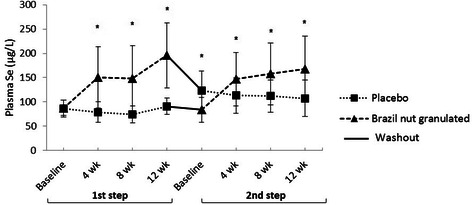
Plasma Se levels during intervention with Brazil nut, placebo and washout period. Data are expressed as the mean ± SD. Student’s *t*-test between placebo and Granulated Brazil nut groups. * *p* < 0.05

Basal plasma Se concentration from the study group was 87.0 ± 16.8 μg/L and it was observed that 57.1 % (*n* = 52) presented low plasma Se (<90 μg/L). Throughout intervention, Se plasma concentrations increased significantly with the *Brazil nut intake (Fig. 2). It has been observed an overall increase in Plasma Se by 119 %. However, it was possible to verify the carry over effect after the washout period on placebo diet Se concentrations; this occurred because part 112of the study group who received Granulated Brazil nut supplementation on the first steps did not return to the same basal values, making plasma Se initial value to be elevated in relation to Granulated Brazil nut supplementation initial value.

Significant increase of GPx3 activity and oxidized-LDL reduction with Granulated Brazil nut intake was observed (Table 2). At the end of 12 weeks plasma total antioxidant capacity was lower on the placebo diet than on the Brazil nut diet (*p* = 0.024).

**Table 2:** The effect of partially defatted Granulated Brazil nut on antioxidant status and oxidative stress markers in hypertensive and dyslipidemic patients

	Diet + placebo		Diet + Granulated Brazil nut	
	Before	12 weeks	Before	12 weeks
GPx3 (nmol/min/mL)	107.53 ± 27.79	115.06 ± 38.09 ^a^	112.66 ± 40.09 ^b^	128.32 ± 38.31 ^b, a^
TAC (%)	20.36 ± 4.44	18.75 ± 5.64 ^c^	20.13 ± 4.68	20.41 ± 4.80 ^c^
8-epi PGF_2α_ (pg/mL)	14.57 ± 7.84 ^d^	16.37 ± 9.81^d^	15.83 ± 9.50	15.65 ± 8.08
Oxidized LDL (U/L)	64.43 ± 20.97	63.76 ± 23.03	66.31 ± 23.59 ^e^	60.68 ± 20.88 ^e^
Ox LDL /LDL-c	0.56 ± 0.18	0.57 ± 0.19	0.57 ± 0.18	0.53 ± 0.15

Mean ± SD. ^a, b, c, d, e^ p < 0.05. Means followed by the same alphabetic letter are statistic different. Paired Student’s *t*-test (^b, d, e^) and Student’s *t*-test (^a, c^). GPx3: plasma glutathione peroxidase; TAC: total antioxidant capacity of plasma; 8-epi PGF_2α_: 8-epi prostaglandine F_2α_; LDL: low density lipoprotein; Ox LDL: oxidized low density lipoprotein

**Fig. 3:**
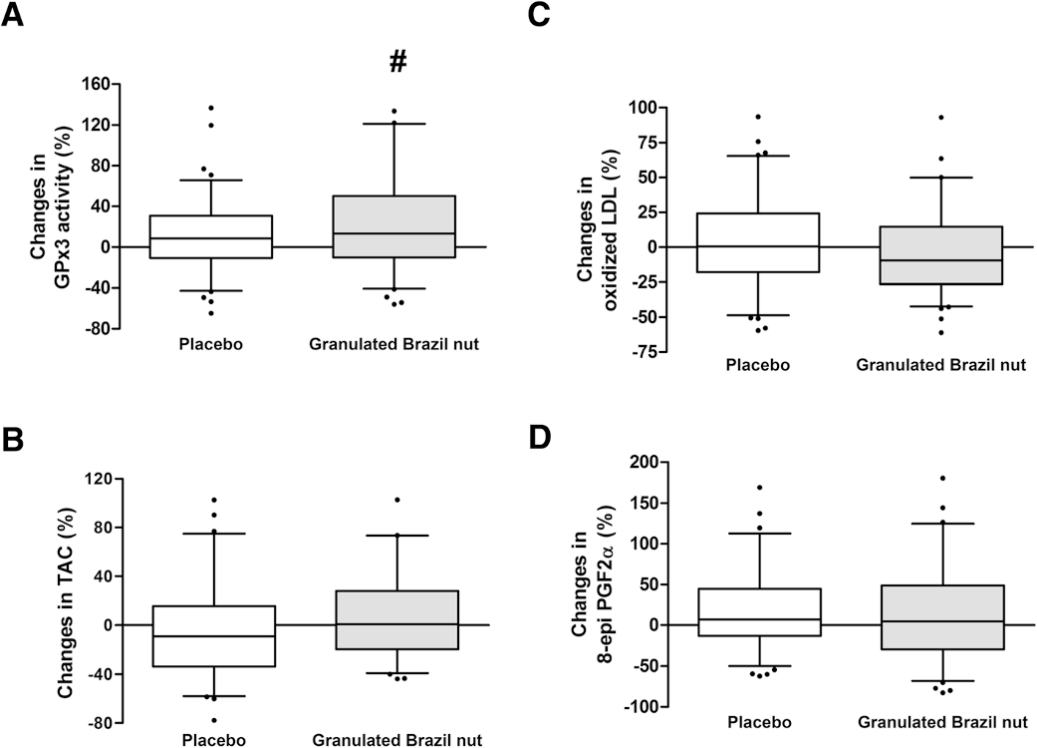
Changes at 12 weeks in the markers of antioxidant status and oxidative stress. # p < 0.05. Student’s *t*-test (**a**, **b**, **c**, **d**). GPx3: plasma glutathione peroxidase; TAC: total antioxidant capacity of plasma; 8-epi PGF_2α_: 8-epi prostaglandin F_2α_; LDL: low density lipoprotein

The oxidative stress markers variation percentage and antioxidant capacity according to dietary intervention at the end of the study is presented in Fig. 3. It was observed increase in GPx3 activity by 24.8 % in Granulated Brazil Nut and it was statistical different from placebo (*p* = 0.034), an oxidized-LDL reduction by 3.2 % after Granulated Brazil Nut intervention but it was no statistical different from placebo. There were no statistical difference between Granulated Brazil Nut and placebo for plasma total antioxidant capacity and 8-epi PGF2α (Fig. 3).

**Fig. 4:**
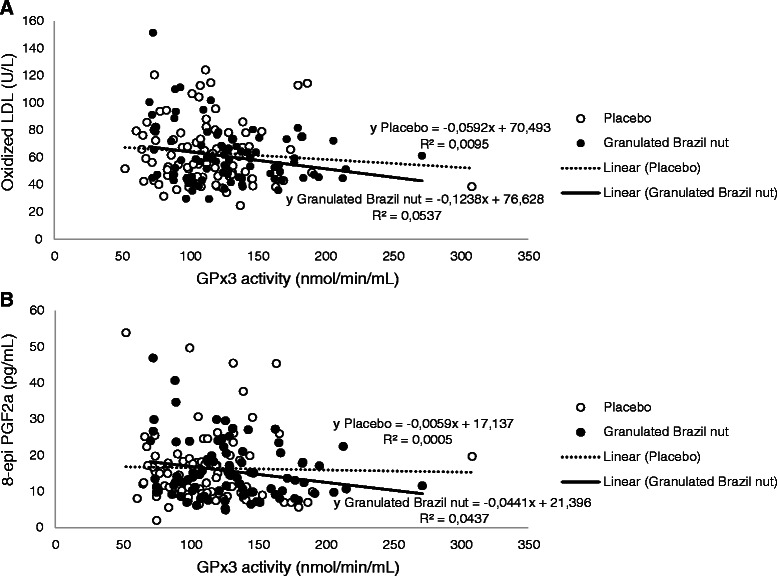
Association between GPx3 activity and biomarkers of oxidative stress. Simple linear regression. **a**. Granulated Brazil nut (*p* = 0.032) and Placebo (*p* = 0.371); **b**. Granulated Brazil nut (*p* = 0.052) e Placebo (*p* = 0.836)

A significant inverse association between oxidized-LDL and GPx3 activity after Granulated Brazil nut intake is shown in Fig. 4 with a simple linear regression model (B − 0.434, IC 95 % − 0.829;−0039, β − 0.232, *p* = 0.032) and it remained significant after adjustment for age, BMI, gender, diabetes and smoking status (B − 0.556, IC 95 % − 0.963;−0148, β − 0.298, *p* = 0.008). However no statistical significance was observed between 8-epi PGF2α and GPx3 activity with the simple linear regression model (B − 0.991, IC 95 % − 1.991;−0009, β − 0.209, *p* = 0.052) as shown in Fig. 4. Nevertheless, a significant inverse association between 8-epi PGF2α and GPx3 activity was achieved after adjustment for age, BMI, gender and diabetes (B − 1.082, IC 95 % − 2.081;−0.084, β − 0.234, *p* = 0.034).

## Discussion

The present study showed that the partially defatted Granulated Brazil nut (13 g/day, providing 227.5 μg) in a serum lipid and blood pressure reduction diet for dyslipdemic and hypertensive patients, increased antioxidant status and reduced oxidative stress. The relationship of these effects were inversely proportional.

The Brazil nut also has an important amount of antioxidants compounds, such as almonds, hazelnuts, macadamia nuts, pine cones, and cashew nuts [36]. The Brazil nut is the food with the highest amount of Se in its composition [1]. Se is a mineral which is involved with the protection against oxidative stress via selenoproteins as glutathione peroxidase, participanting of this enzyme active site [29]. Besides, Brazil nut is high in β-tocopherol (88.3 % of total tocopherols), a potent antioxidant [37].

Thomson *et al.* demonstrated that the Brazil nut intake significantly improved GPx activity on individuals with low plasma Se compared to selenomethionine or placebo [4]. SELGEN study measured the Se status of healthy volunteers before and after supplementation with 100 μg Se/day for six weeks followed by a washout period of six weeks, and the authors showed a rapid and significant decrease in plasma Se status to baseline levels after two weeks and continued to fall at fourth week after which time it was similar to baseline concentration [20]. However in the present study there was a carryover effect, maybe because of the selenium dose which was two-fold SELGEN study dose, and the main pools of Se in human body are selenoproteins and selenomethionine [38].

Many enzymatic processes can generate ROS on the human body, and there are evidences that connect these processes to cardiovascular diseases pathophysiology [12]. The lipid peroxidation process is initiated by a ROS reaction with a unsaturated fatty acid and propagated by peroxyl radicals [39]. Glutathione peroxidase biochemical function is reducing lipid hydroperoxides to their correspondent alcohols and decreasing free hydrogen peroxide to water [40]. GPx1 enzyme (eritrocity) activity was reduced in CAD patients and low activity of this enzyme was a predictor to cardiovascular event in five-year’s time [41]. Similar to the present study, other studies also observed GPx activity increase after Brazil nut supplementation in obese women [42] and CKD patients in hemodialysis [43]. In addition, it has been observed that the overall increase in Plasma Se and plasma GPx activity was higher than Thomson *et al.* showed with a low plasma Se subjects [4].

The oxidation of LDL was increased in the familial hypercholestolemia, hypertriglyceridemia, diabetes and coronary artery disease [44]. Clinical trials evaluating different types and quantities of nuts indicated improvement of oxidative stress after nuts’ intake [45]. The reduction of average 15 % on oxidized-LDL in hyperlipidemic men and women was observed after almonds intake (37 or 75 g/day) for a month, compared to the control diet [46]. The present study showed a modest significant reduction in oxidized-LDL after Granulated Brazil nut intake, and it can be attributed to the complex health status of these patients related to duration of disease, previous cardiovascular event and poor blood pressure control and serum lipids control, which may have influenced the findings. However, the observation of such reduction has been inversely associated with GPx3 activity increase, demonstrating a beneficial relationship of Brazil nut antioxidants properties as part of hypertensive and dyslipidemic patients’ nutrition intervention.

High concentrations of F2-isoprostanes are associated with the coronary artery disease extension and severity [47]. In other study, the Brazil nut intake (15 a 25 g/day) for four months did not alter urinary 8-epi PGF2α in female obese teenagers [48]. In the same way, 13 g/day Granulated Brazil nut consumption in the present study did not alter 8-epi PGF2α plasma concentration after twelve week, but an inverse correlation of this biomarker was observed with GPx3 activity, however it was not significant.

The present study has limitations that may have influenced the findings. Such limitations include the cohort of participants who did not complete the study whose age was significantly less than those who did complete the study, the relative short intervention period, the heterogeneity of the participants, and the carry-over effect in relation to plasma selenium concentrations. However, the crossover design allows the patient to be their own control. Finally, partially defatted Granulated Brazil nut are easy to include in one’s diet and use in daily recipes.

The present study contributes to the rising evidence which demonstrates the beneficial effects of nuts over CVD risk factors. Our data showed that Granulated Brazil nut can be recommended as part of a healthy diet for the heart, since it has a potential benefit to increase plasma selenium, increase enzymatic antioxidant activity of GPx3 and to reduction oxidation in LDL in hypertensive and dyslipidemic patients.

## Abbreviations

ACE: Angiotensin-converting enzyme
AMI: Acute myocardial infarction
ASA: Acetylsalicylic acid
BMI: Body mass index
CABG: Coronary artery bypass grafting
CHD: Coronary heart disease
DBP: Diastolic blood pressure
HDL: High-density lipoprotein
LDL: Low-density lipoprotein
SBP: Systolic blood pressure
Se: Selenium
WC: Waist circumference
GPx3: Plasma glutathione peroxidase
TAC: Total antioxidant capacity of plasma
8-epi PGF_2α_: 8-epi prostaglandine F_2α_
